# Enhancing weld performance of AA2024-T351 using drilled-to-bossed geometry and deep learning prediction in direct drive friction welding

**DOI:** 10.1038/s41598-026-52231-1

**Published:** 2026-05-10

**Authors:** Eyob Messele Sefene, Alemayehu Admas Abay, Akshansh Mishra, Assefa Asmare Tsegaw, William Gomez, Addisalem Desalegn Sisay, Ajay Kumar

**Affiliations:** 1 Department of Mechanical Engineering, National Taiwan University of Science and Technology, No. 43, Keelung Rd, Sec.4, Da’an Dist, Taipei, 10607 Taiwan; 2 Mechanical Engineering Department, Debre Berhan University, P.O. box 445, Debre Berhan, Ethiopia; 3 School of Industrial and Information Engineering, Politecnico Di Milano, Milan, Italy; 4 Faculty of Mechanical and Industrial Engineering, Bahir Dar Institute of Technology, Bahir Dar University, P.O. Box 26, Bahir Dar, Ethiopia; 5 Department of Industrial Management, National Taiwan University of Science and Technology, Taipei City, 106335 Taiwan; 6 Institute of Digital Learning and Education, National Taiwan University of Science and Technology, Taipei, Taiwan; 7 Department of Mechanical Engineering, School of Engineering, Faculty of Science, Technology and Architecture, Manipal University Jaipur, Jaipur, Rajasthan 303007 India

**Keywords:** Direct drive friction welding, AA2024-T351, Torsional tensile strength, Tensile strength, Machine learning, Prediction, Engineering, Materials science

## Abstract

The growing demand for lightweight, high-strength materials in the aerospace and automotive industries has brought aluminium alloys such as AA2024-T351 into the spotlight due to their outstanding strength-to-weight ratio. However, achieving reliable joints in such alloys remains a significant challenge, particularly when using conventional welding techniques. To address this, the present study focuses on optimizing process parameters in Direct Drive Friction Welding (DDFW) for AA2024-T351. A novel drilled-to-bossed geometry, inspired by the traditional mortise-and-tenon joint, is introduced to enhance mechanical interlocking and improve weld integrity. Experimental trials were systematically designed using an L_18_ orthogonal array to evaluate both tensile and torsional strength. The experiments were conducted on a customized engine lathe, with friction pressure, forging pressure, spindle speed, faying surface geometry, and friction time selected as the controllable process parameters. In parallel, advanced deep learning models, including an ensemble residual network, a compact attention network, and an adaptive multiscale network, were implemented to predict ultimate tensile strength based on the welding conditions. The results revealed that the optimal parameter combination of 30 MPa friction pressure, 70 MPa forging pressure, 2200 rpm spindle speed, drilled-to-bossed geometry, and 4 min of friction time yielded exceptional mechanical performance, achieving a tensile strength of 538 MPa and torsional strength of 325 MPa. The drilled-to-bossed configuration demonstrated significantly higher joint strength compared to conventional flat-to-flat joints. Moreover, among the deep learning models, the ensemble residual network achieved the highest predictive accuracy with an R² value of approximately 0.81, effectively capturing the complex relationship between process parameters and weld strength.

## Introduction

**Fig. 1:**
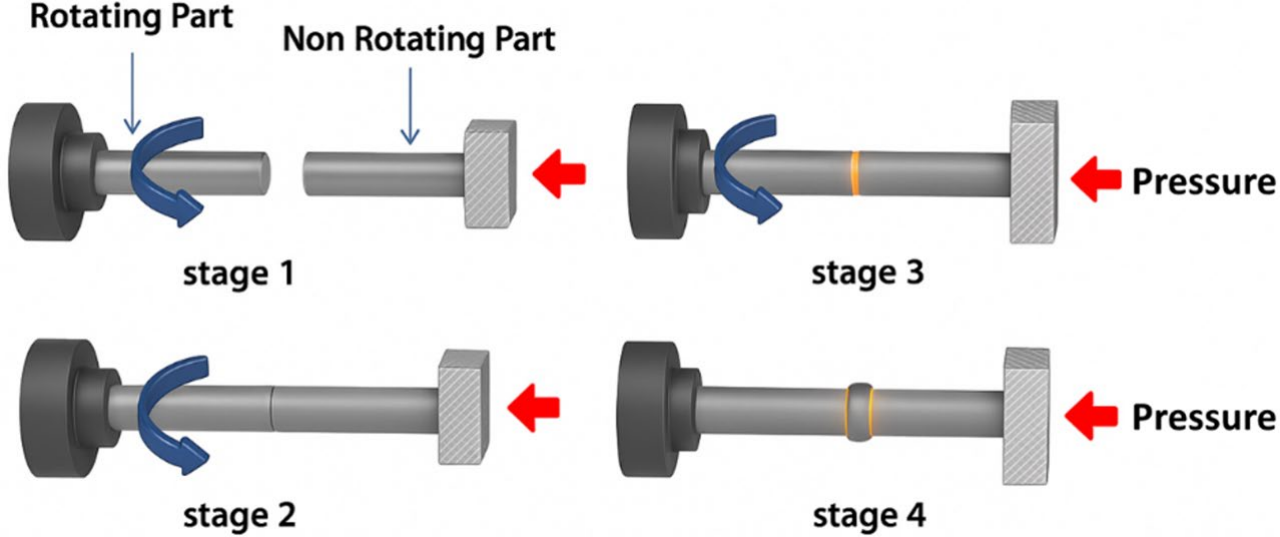
Stages of friction welding process.

 The demand for advanced joining technologies has intensified in recent decades, driven by the accelerated growth of aerospace, automotive, and marine industries^1,2^. These sectors require structural materials that combine lightweight characteristics with exceptional mechanical integrity and corrosion resistance. Aluminum alloys, particularly the AA2024-T351 grade, are among the most widely used aerospace-grade materials due to their excellent strength-to-weight ratio and fatigue resistance. However, these same properties render them notoriously difficult to join using conventional fusion welding methods, due to issues such as hot cracking, porosity, and the degradation of joint performance^3–5^. In contrast, solid-state welding techniques, which circumvent the melting of base materials, offer a transformative solution for joining such challenging alloys. These processes promote coalescence through atomic diffusion and severe plastic deformation, typically under elevated pressure and moderate temperatures well below the melting point of the materials involved^6–8^. Among the various solid-state approaches, friction welding (FW) has emerged as one of the most efficient and reliable methods for producing defect-free, high-strength joints without the need for filler materials or shielding gases^9,10^. A key subset of friction welding, direct drive friction welding (DDFW), has shown substantial promise for joining precipitation-hardened aluminum alloys such as AA2024-T351. The DDFW process involves the continuous rotation of one workpiece against a stationary counterpart under controlled axial pressure. As frictional heat builds up at the interface, the material softens and undergoes plastic deformation. Once the desired thermal condition is achieved, rotation is halted, and a forging load is applied to complete the bond. The schematic stages of this process from initial rubbing to flash formation are illustrated in Fig. 1. This precise mechanical control enables reproducible weld quality with minimal heat-affected zones (HAZ), a critical factor in high-performance applications^11–13^. In DDFW, the synergistic effects of time, temperature, and axial pressure facilitate the coalescence of base materials without significant melting, thereby preserving the intrinsic properties of the parent metals^14^. Optimizing the process parameters in DDFW is essential for enhancing weld joint strength, structural integrity, and long-term reliability in demanding applications. Despite its proven advantages, there remains a significant gap in comprehensive studies that leverage advanced techniques, particularly machine learning for systematic parameter optimization in DDFW.

Recent developments in artificial intelligence and data-driven modeling offer promising avenues for predictive and adaptive control of key process variables such as friction time, axial pressure, and rotational speed. In the following sections, we critically review key contributions from the literature addressing the optimization of DDFW parameters. Shanjeevi et al.^15^ investigate the optimization of friction welding parameters for joining dissimilar materials using a rotary friction welding (RFW) process, focusing on achieving high joint strength through experimental design and regression modelling. The study aims to determine the best set of parameters, like friction pressure, upset pressure, and friction time for maximizing joint tensile strength in dissimilar material pairs. Results show that under optimal conditions (friction pressure ~ 15 MPa, upset pressure ~ 21–24 MPa, friction time ~ 5–7 s), joints achieved maximum tensile strength (~ 12 kN, approximately 189 MPa) with ~ 92% joint efficiency. Impact toughness improved with higher friction pressure, and plastic deformation increased near the weld interface; microhardness profiles varied across the aluminum and steel sides of the joint. Kunhirunbawon et al.^16^ investigate the optimization of rotary friction welding parameters for aluminum alloy AA6063 using an Adaptive-Network-Based Fuzzy Inference System (ANFIS). The study aims to accurately predict ultimate tensile strength (UTS) from inputs including rotational speed, welding time, and friction pressure, enabling automated control in smart factory settings. Results demonstrate that the ANFIS model effectively predicts UTS from the chosen parameters, facilitating parameter adjustment for automatic process control, thus enhancing joint quality and efficiency. Liu et al.^17^ investigate the prediction of joint upset (axial shortening) in inertia, linear, and continuous-drive friction welding processes using finite element simulation paired with a radial basis function (RBF) neural network. The study aims to provide a robust predictive model for final upset displacement based on welding parameters like initial rotational speed and axial pressure. Results show that the RBF network achieved high prediction accuracy, with correlation coefficients of ~ 0.998 for continuous-drive friction welding and ~ 0.963 for linear friction welding, and less than 8.2% error compared to simulation outputs. Buffa et al.^18^ investigate weld quality in linear friction welding (LFW) of AA6082-T6 aluminum alloy, aiming to predict whether a solid sound weld will form, based on interfacial temperature and strain evolution. The study applies an integrated numerical tool combining finite element analysis and bonding criteria to forecast zones of sound bonding, overheating, or lack of weld. Results show that the model accurately distinguishes between “no weld,” “sound weld,” and “excess heat” zones, matching experimental observations. It predicts peak temperature and strain distributions that determine bonding quality, helping to optimize welding parameters and avoid defects. S et al.^19^ investigate the prediction and optimization of tensile strength in friction-welded joints between SA213 tubes and SA387 tube plates, using a friction welding tube-plate external tool (FWTPET) process. The study combines experimental trials with variables such as rotational speed, tube projection, depth of cut, and presence of a backing block and optimization techniques including Taguchi design, ANOVA, and genetic algorithms. Results show that using a backing block and interference fit without a hole in the tube yielded the highest tensile strength (~ 836.8 MPa), compared to ~ 762.2 MPa without a backing block. Predicted strengths closely matched experimental values under optimal conditions (1300 rpm, 1 mm projection, 0.5 mm depth), while grain refinement around the weld zone contributed to enhanced weld integrity. Hassan et al.^20^ investigate the influence of forge pressure under varying friction times on the metallurgical and mechanical properties of rotary friction-welded AISI 316 stainless steel joints. The study aims to identify optimal conditions for achieving high-performance joints with enhanced tensile strength, ductility, and microstructural integrity. Results show that applying a forge phase reduced flash formation by ~ 30% and refined the heat-affected microstructure. Barrionuevo et al.^21^ investigate the use of laser-assisted rotary friction welding (LA-RFW) to join AISI 1045 steel and 2017-T4 aluminum alloy, aiming to improve joint performance and predict ultimate tensile strength (UTS) using machine learning models. The study compares the predictive capability of machine learning with response surface methodology (RSM). Results show that ML models offer superior accuracy in predicting UTS, while laser assistance enhances interface bonding and increases joint strength. Khosrowshahi et al.^22^ investigate the plastic deformation behavior during direct-drive friction welding of AISI 4140 and ASTM A106 steel tubes using numerical (finite element) simulation, aiming to predict stress–strain distribution and microstructural evolution under different welding parameters. Results show that the simulation accurately captures deformation zones and strain localization patterns in both steels, providing detailed insight into heat generation and plastic flow without experimental trials. Aoki et al.^23^ investigate the effect of applied pressure during linear friction welding (LFW) of quenched-and-tempered SCM440 martensitic steel, aiming to understand how pressure influences joining temperature, microstructure, and hardness distribution. Results show that increasing pressure from 150 to 900 MPa lowers the joining temperature into the intercritical phase range leading to a two-phase martensite ferrite microstructure and a reduced maximum hardness (~ 552 HV). At even higher pressure (1200 MPa), an overshoot in local heat raises temperature above the A₃ point, re-forming fine fully martensitic microstructures (~ 772 HV) and narrowing the hardened zone to ~ ± 0.10 mm, compared to ~ ± 0.38 mm at 150 MPa. Hardness softening decreases and the soft zone hardness increases with pressure. This study introduces a novel joint design strategy by comparing drilled-to-bossed and flat-to-flat interface configurations to enhance and evaluate weld joint efficiency. Both tensile and torsional strengths are considered as primary response variables to assess joint performance. To accurately predict tensile strength, the study employs advanced deep learning models, including compact attention networks, ensemble residual architectures, and adaptive multiscale learning frameworks. The results demonstrate that the drilled-to-bossed configuration significantly improves weld joint strength compared to the conventional flat-to-flat approach, offering a promising solution for structural applications requiring enhanced mechanical performance.

## Experimental setup and methodology

**Fig. 2:**
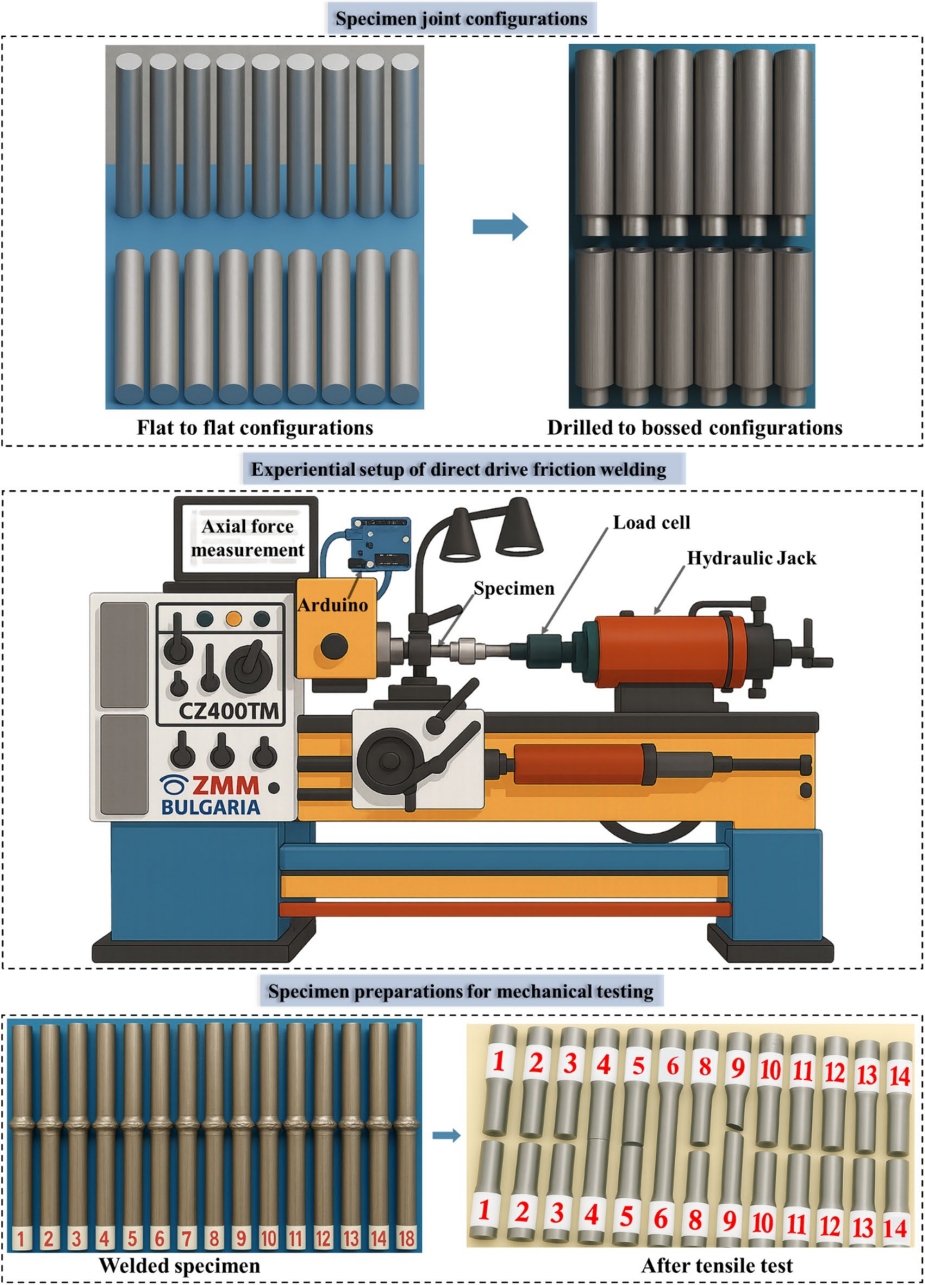
Experiential setup for direct drive friction welding.

**Fig. 3:**
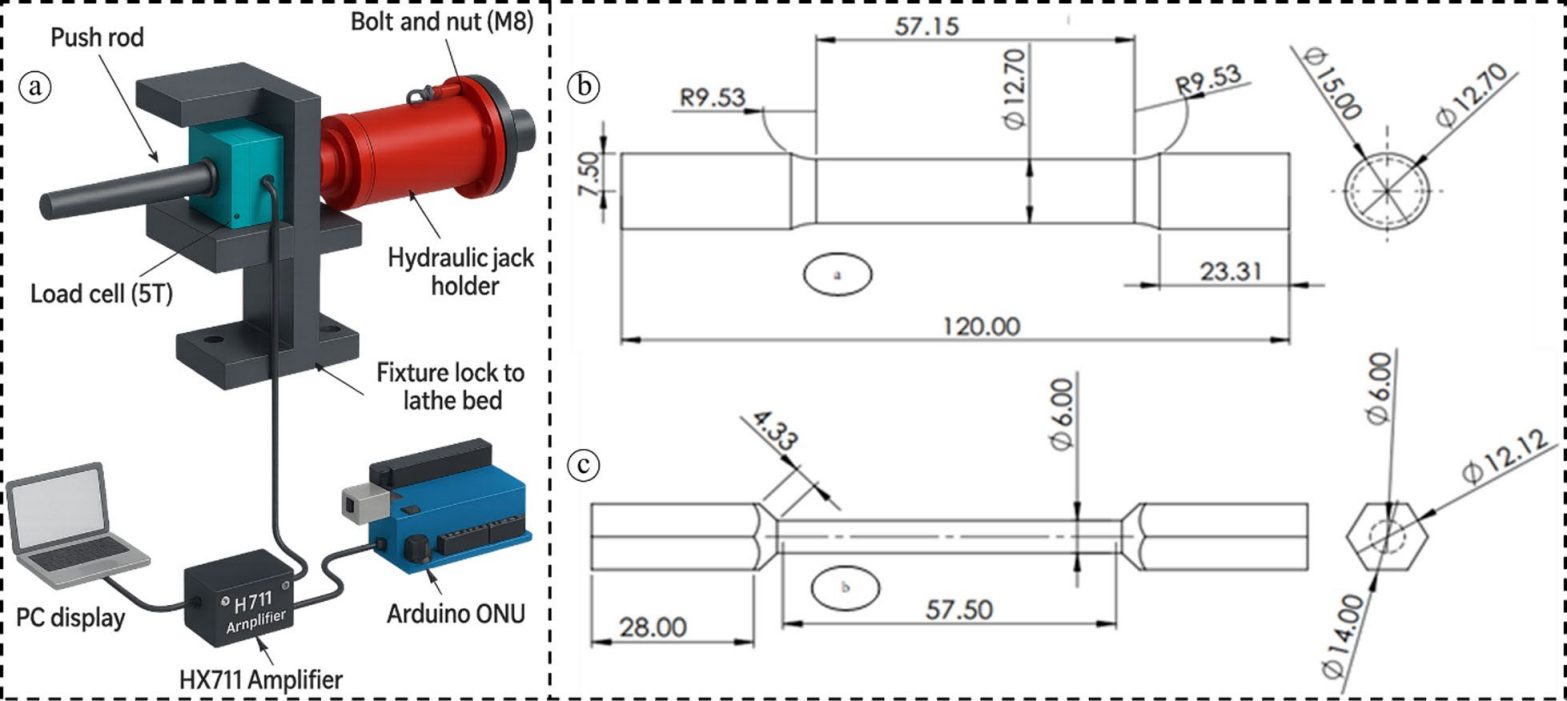
**a** axial force measurement system, **b** ASTM E8-04 standard for the tensile test, and **c** ASTM-F383 standard for the torsional test^25^.

In this study, AA2024-T351 aluminum alloy was selected as the base material in a butt joint configuration. Its detailed chemical composition and mechanical properties are presented in Tables 1 and 2, respectively. Cylindrical specimens with dimensions of Ø15 mm × 60 mm were prepared in accordance with standard tensile and torsional testing specifications. Friction welding was performed using a customized ZMM (Bulgaria) engine lathe, modified to accommodate the experimental setup. A total of 36 specimens were welded following the Taguchi L_18_ orthogonal array to evaluate the influence of process parameters on joint performance. A total of 36 base metal specimens were prepared, resulting in 18 welded joints, since each joint was produced by welding two cylindrical specimens together. All 18 experimental runs defined in the L_18_ orthogonal array were successfully completed. No runs were excluded, and all fabricated joints were tested and included in the reported analysis. Each experimental condition generated one welded joint corresponding to a specific combination of process parameters. After welding, the joints were prepared as required and subjected to both tensile and torsional testing. The measured tensile strength and torsional strength values were recorded for each run and treated as the multi-objective response variables in the optimization and performance evaluation. To enhance weld joint strength, two novel butt joint configurations, drilled-to-boss and flat-to-flat, were developed, as depicted in Fig. 2. The controlled process parameters included faying surface geometry, friction pressure, forging pressure, rotational speed, and friction time, with corresponding levels summarized in Table 3. A secondary tailstock was employed to support the hydraulic jack fixture on the lathe. The force exerted by the hydraulic jack, according to each experimental condition, was measured using a compression-type load cell and regulated by an Arduino UNO REV3 integrated with an HX711 amplifier, as shown in Fig. 3. The tensile and torsional strengths of the welded joints considered as multi-objective response variables were assessed using a universal testing machine (Model: HUT-300) and a torsional test device (Model: HSM31), respectively.

**Table 1:** Chemical composition of AA2024-T351^24^.

Alloy	Si	Fe	Cu	Mn	Mg	Cr	Zn	Ti
AA2024-T351	0.08	0.17	4.5	0.7	1.54	< 0.01	0.06	0.03

**Table 2:** Mechanical properties for AA2024-T351 aluminum alloy^24^.

Mechanical properties	Standard values
Ultimate tensile strength (MPa)	540
Yield tensile strength (MPa)	324
Shear strength (MPa)	330
Fatigue strength (MPa)	138
Modulus of elasticity (GPa)	73.1
Shear modulus (GPa)	28

**Table 3:** Parameters and their level settings.

Parameters	Levels		
	1	2	3
Contact/ faying geometry	Flat-to-flat	Drilled-to-bossed	–
Friction pressure (MPa)	20	30	40
Forging pressure (MPa)	30	50	70
Rotational speed (rpm)	1120	1580	2240
Friction time (min)	2	3	4

### Drilled-to-bossed joint configuration

**Fig. 4:**
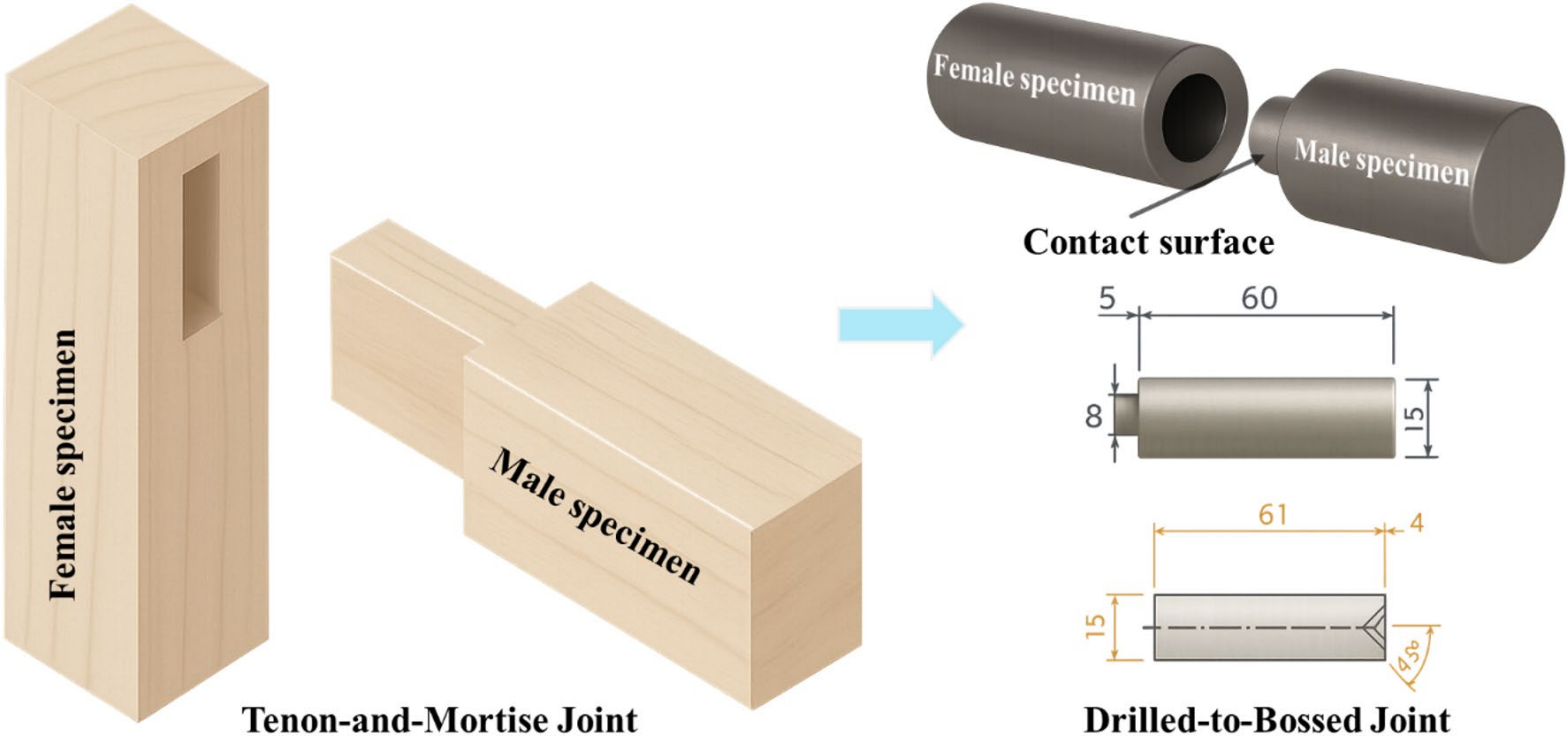
Development of drilled-to-bossed joint inspired by mortise-and-tenon design.

In conventional friction welding, the flat-to-flat butt joint configuration is predominantly employed owing to its simplicity in preparation and alignment. However, this configuration often provides limited mechanical interlocking at the interface, which can reduce weld joint efficiency under complex loading conditions. To overcome this limitation and improve joint integrity, we draw inspiration from the traditional tenon-and-mortise joint renowned in woodworking and structural engineering for its superior stability and load transfer, and introduce a novel drilled-to-bossed configuration, as illustrated in Fig. 4. The drilled-to-bossed configuration comprises a male specimen with a protruding boss and a female specimen with a centrally drilled cavity. During the friction welding process, the boss of the male specimen engages with the drilled cavity of the female counterpart, thereby increasing the effective contact surface area and establishing a robust mechanical interlock at the weld interface. The male specimen was designed with a cylindrical boss of 8 mm diameter and 60 mm length, while the female specimen contained a corresponding drilled hole with a nominal diameter of 8 mm to accommodate the boss. To ensure consistent contact conditions and reproducibility of the joint configuration, all geometrical features were machined using precision turning and drilling operations, maintaining a dimensional tolerance of approximately ± 0.02 mm for both the boss and the drilled cavity. A slight clearance fit was intentionally adopted between the boss and the drilled hole to facilitate accurate alignment of the specimens prior to welding while avoiding excessive interference that could hinder proper assembly. This controlled clearance enables stable initial contact during the friction stage and promotes uniform heat generation and plastic deformation at the interface. To ensure repeatability, the geometric preparation of all specimens was performed using the same machining procedure and inspection protocol before welding. This interlocking mechanism acts as a geometric reinforcement, distributing the applied load more uniformly and minimizing the risk of interface slippage or debonding under axial and torsional stresses. Furthermore, the enhanced interfacial constraint promotes more efficient plastic deformation and heat generation during the friction phase, resulting in stronger metallurgical bonding. Integrating this configuration into the DDFW process is expected to significantly improve tensile and torsional strength compared to the conventional flat-to-flat design. These improvements are primarily attributed to the increased fusion zone area and the mechanical constraint provided by the interlocking geometry.

### Deep learning algorithms used in the present work

#### Data preparation

**Fig. 5:**
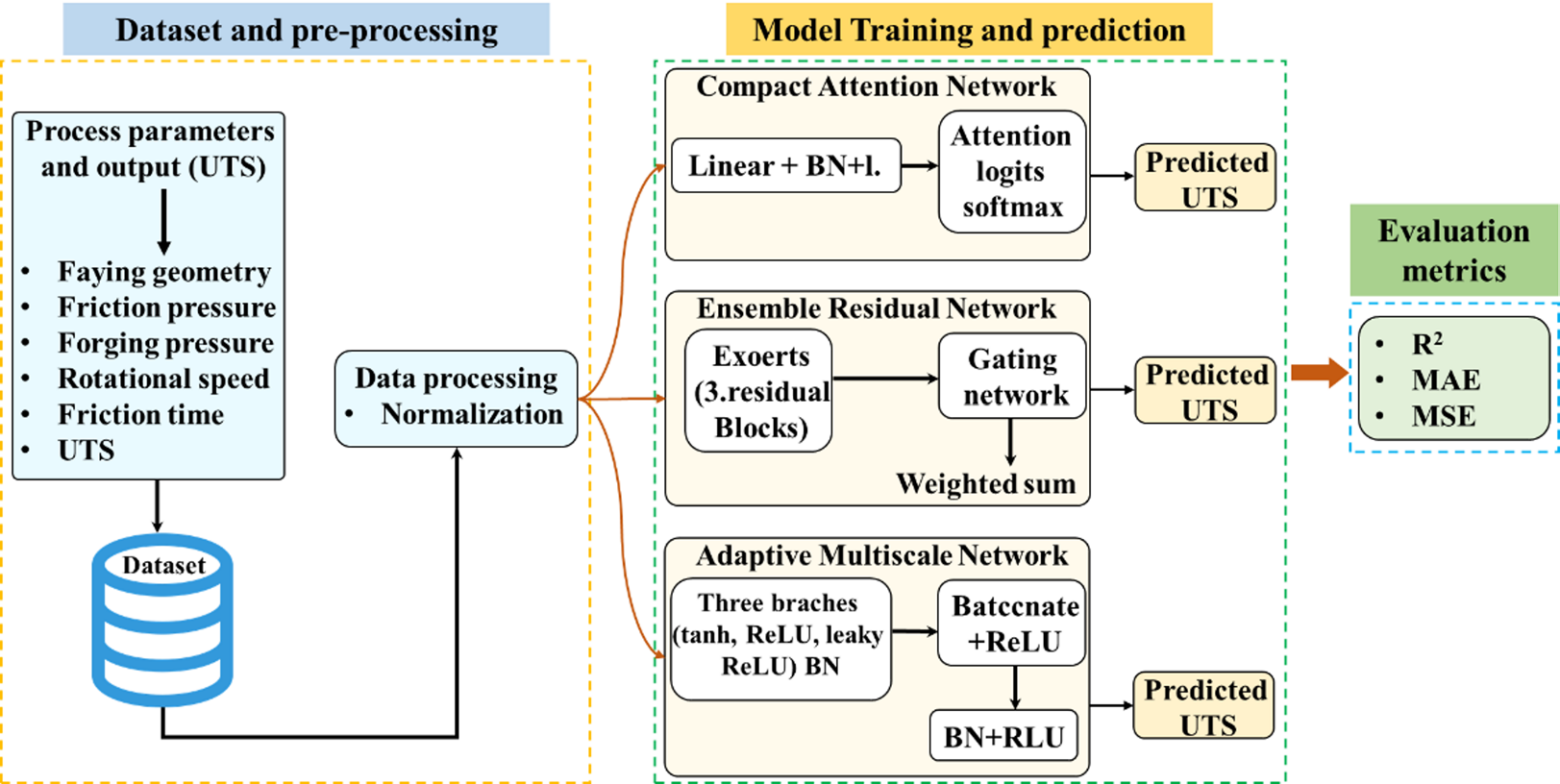
Prediction framework using Deep learning models.

The input dataset for model development consisted of the DDFW process parameters and derived features corresponding to each welding experiment. Five primary process variables were considered: faying surface geometry (categorical: flat-to-flat vs. drilled-to-bossed), friction pressure, forging pressure, spindle rotational speed, and friction time. To enrich the model’s predictive capacity, additional engineered features were formulated from these variables. In particular, a pressure ratio (forging pressure to friction pressure) and an energy index (a combined measure reflecting heat input, based on friction pressure and friction time) were calculated, along with an interaction term capturing the coupled effect of rotational speed and friction pressure. These engineered features introduce nonlinear combinations of the process parameters, providing the models with insight into complex interdependencies that a purely linear model might miss. All features were numerically scaled and normalized to ensure commensurate ranges and to facilitate stable network training. Continuous variables were standardized (approximately zero-mean, unit-variance), yielding both positive and negative normalized values across the dataset. The categorical faying surface geometry was one-hot encoded as a binary indicator (0 for flat-to-flat, 1 for drilled-to-bossed), allowing the networks to learn the influence of joint configuration. This careful feature engineering and normalization help expose underlying patterns to the deep learning models and mitigate bias from scale differences, thereby laying a strong foundation for accurate UTS prediction. The overall deep learning prediction framework has been illustrated in Fig. 5. All deep learning models were implemented in Python 3.10 using PyTorch 2.0 and scikit-learn 1.3, executed on a standard CPU environment. The EnsembleResidual, AdaptiveMultiScale, and CompactAttention architectures were each optimized using the AdamW optimizer with a cosine annealing learning rate scheduler (initial learning rate: 3 × 10⁻³ to 5 × 10⁻³, minimum: 1 × 10⁻⁵, weight decay: 1 × 10⁻⁴). Dropout rates were set in the range 0.15–0.25 and batch normalization was applied after each hidden layer to mitigate overfitting given the small sample size (*n* = 18). Gradient clipping (max norm = 1.0) was employed to ensure training stability. Hyperparameters including hidden layer width (32–128 units), dropout rate, learning rate, and maximum epochs (500–700) were selected through manual search guided by LOOCV validation loss, as the limited dataset size precluded computationally intensive strategies such as grid or Bayesian optimization. Early stopping with a patience of 70–90 epochs was applied to prevent overfitting, with the final model selected based on the lowest mean squared error (MSE) observed across LOOCV folds. Input features were standardized using zero-mean unit-variance scaling via scikit-learn’s StandardScaler, applied independently within each LOOCV fold to prevent data leakage. Random seeds were fixed (NumPy: 42, PyTorch: 42) throughout all experiments to ensure full reproducibility.

##### Compact attention network architecture

The Compact Attention Network (CAN) was designed to efficiently capture the relative importance of each feature in predicting UTS. This model begins by projecting the normalized input feature vector $\:x\in\:{\mathbb{R}}^{d}$x (with $\:d$ being the number of input features, here $\:d=8$) into a higher-dimensional hidden representation through a linear transformation, followed by batch normalization and a non-linear activation (ReLU). This can be expressed as a hidden vector in Eq. (1)^26^.

1 $$\:h=ReLU\left(BatchNorm\right({W}_{1}x+{b}_{1}\left)\right)$$

where $\:{W}_{1}$​ is the weight matrix for feature projection and $\:{b}_{1}$​the bias term. The result $\:h$ is a rich, learned feature representation of the input welding parameters in the hidden space. Next, the network implements a compact attention mechanism to focus on the most salient features in $\:h\:$for UTS prediction. Rather than employing a full attention matrix (which would be excessive for the small input size), the CAN computes a single attention weight for each hidden feature dimension. This is done by passing $\:h$ through a second linear layer to produce attention logits, and applying a softmax normalization to obtain the attention weight vector $\:\alpha\:$ as expressed in Eq. (2)^27^.

2 $$\:\alpha\:=softmax({W}_{2}h+{b}_{2})$$

Here $\:{W}_{2}$ and $\:{b}_{2}$are the trainable parameters of the attention-generating layer, and $\:\alpha\:\in\:{\mathbb{R}}^{m}$ (with $\:m$ equal to the hidden dimension) contains non-negative weights that sum to 1. Each component $\:{\alpha\:}_{i}$ indicates the relative importance of the corresponding hidden feature $\:{h}_{i}\:$for the prediction. The hidden representation is then re-weighted by these attention scores via element-wise multiplication, yielding an attended feature vector in Eq. (3)^28^.

3 $$\:{h}_{att}=h\odot\:\alpha\:,$$

where ⊙ denotes element-wise multiplication. This operation amplifies the influence of critical features and suppresses less relevant ones, effectively directing the network’s “attention” to the most informative aspects of the welding conditions. Finally, the attended feature vector $\:{h}_{att}$ is fed into a regression head composed of fully connected layers (with ReLU activations) to map the weighted features to the target output. In practice, two dense layers were used in sequence, followed by a linear output neuron that produces the predicted UTS value $\:y.$ The overall architecture of the Compact Attention Network thus consists of an input projection layer, an attention-weighting mechanism on the hidden features, and a multi-layer perceptron output stage. This design enables the model to achieve a balance between complexity and interpretability: it is compact enough to avoid overfitting on a small dataset, yet capable of feature prioritization through its attention weights, which improves learning of the complex parameter UTS relationships. Notably, the distributed pattern of learned attention weights (spanning roughly 0.02–0.06 in the case study) indicates that the network avoids over-concentrating on any single feature, instead combining cues from multiple parameters for robust prediction.

##### Ensemble residual network architecture

The Ensemble Residual Network (called *EnsembleResidualNet*) was developed to improve generalization by integrating multiple predictive sub-models with residual learning^29,30^. In this architecture, several parallel “expert” networks $\:{f}_{i}\left(x\right)$ are trained on the same input features, and their outputs are combined in an adaptive, weighted manner. Each expert subnetwork is a feed-forward neural network that includes an internal residual connection. Specifically, for an input $\:x$, an expert’s forward pass can be written as in Eq. (4)^31^.

4 $$\:{f}_{i}\left(x\right)=\varnothing\:({W}_{2i}.\varnothing\:\left({W}_{1i}x+{b}_{1i}\right)+\varnothing\:({W}_{1i}x+{b}_{1i})$$

where $$\:\varnothing\:$$(⋅) is a non-linear activation function (ReLU), $$\:{W}_{1i}$$, $$\:{b}_{1i}$$​ and $$\:{W}_{2i}$$ are weight matrices and bias for the $i$-th expert’s layers, and the term in parentheses represents the residual skip connection that adds the intermediate feature$$\:\:{W}_{1i}+{b}_{1i}$$ (pre-activation output of the first layer) to the second layer’s input. This residual mapping helps stabilize training and improve gradient flow, which is especially beneficial given the limited size of the dataset. Each expert thus learns a slightly different representation of the input-output mapping, and the residual links enable deeper effective capacity without overfitting. To combine the experts, the EnsembleResidualNet employs a learnable gating network. The gating network takes the input $$\:x$$ and produces a weight for each expert $$\:{f}_{i}$$. These weights are obtained via a softmax function to ensure they form a convex combination (summing to 1) in Eq. (5).

5 $$\:\sum\limits_{i=1}^{N}{g}_{i}\left(x\right)=1,\:\:{g}_{i}\left(x\right)=\frac{{e}^{{z}_{i}}}{\sum\:_{j=1}^{N}{e}^{{z}_{j}}}\:where\:z={W}_{g}x+{b}_{g}$$

With $\:N$ being the number of expert subnetworks, and $\:{w}_{g,}$ $\:{b}_{g,}$ the gating network’s parameters that project the input into a logit vector [ $\:{z}_{1,}\dots\:,\:{z}_{N}$​]. The softmax normalization yields gating coefficients $\:{g}_{i,\left(x\right)}$ that are non-negative and sum to unity, effectively representing the model’s confidence in each expert’s prediction for the given input. The outputs of the expert networks are then aggregated as a weighted ensemble to form the combined hidden representation $\:h$ in Eq. (6).

6 $$\:h=\:\sum\limits_{i=1}^{N}{g}_{i}\left(x\right)\:{f}_{i}\left(x\right).$$

This ensemble output $$\:h$$ (of dimension $h$, the chosen hidden size) is a learned blend of the experts’ intermediate features. By adjusting $g_i(x)$, the model can dynamically emphasize the most relevant expert(s) for each specific welding condition, yielding a form of an input-dependent ensemble. Finally, as in the previous model, the combined representation $$\:h$$ is passed through a prediction head $$\:\psi\:\left(h\right)$$ a small multilayer perceptron that produces the scalar output $$\:y$$ for UTS. In summary, the Ensemble Residual Network effectively acts as a mixture-of-experts model with built-in residual learning. This design is well-suited to capture the multifaceted nature of the welding parameter space: each expert can specialize in certain regimes or patterns (for instance, one expert might excel at low-pressure conditions while another handles high-pressure scenarios), and the gating mechanism learns to select or weight these experts appropriately for each new input. The residual connections within experts ensure that each subnetwork can learn complex functions without vanishing gradient issues, even with limited data. This ensemble approach proved to be highly effective in our case, as it was able to capture the complex relationship between process parameters and UTS more accurately than a single network, ultimately achieving the strongest predictive performance of the three models.

##### Adaptive multiscale network architecture

The Adaptive Multiscale Network was developed to explicitly learn features at multiple scales or levels of abstraction from the input parameters. They have played a significant role in many fields, like computer vision and time series. This architecture processes the input x through multiple parallel transformation branches, each with a different nonlinear activation function, and then fuses these processed features for final prediction^32,33^. The motivation is to allow the model to capture both subtle and pronounced effects of the welding parameters on UTS by simultaneously exploring different feature transformations. Concretely, in our implementation, three parallel feed-forward streams were used: one applying a $\tanh$ activation (to capture saturating or nonlinear trends), one using a standard ReLU (for linear or thresholding behaviors), and one using a leaky ReLU (for handling features across a wider dynamic range). Each branch first applies a linear transformation to the input, followed by batch normalization and its respective nonlinearity. Denoting $$\:{W}_{1},\:{W}_{2},\:{W}_{3}$$​ (and corresponding $$\:{BN}_{1},\:{BN}_{2},\:{BN}_{3}$$ ​) as the weight matrices (and batch norm layers) for the three branches, the transformed outputs are: $$\:{h}^{\left(1\right)}=\mathrm{tanh}\left({BN}_{1}\right({W}_{1x}\left)\right);{h}^{\left(2\right)}=\mathrm{ReLU}\left({BN}_{2}\right({W}_{2x}\left)\right);{h}^{\left(3\right)}=\mathrm{LeakyReLU}\left({BN}_{3}\right({W}_{3x}\left)\right).$$ These branch outputs are concatenated to form a combined feature vector $$\:[{h}^{\left(1\right)}$$; $$\:{h}^{\left(2\right)}$$; $$\:{h}^{\left(3\right)}]$$ that contains a richer representation of the input across different scales. A batch normalization is applied to this concatenated vector for stability, followed by a ReLU activation. The fused multiscale features are then projected back into a lower-dimensional latent space via another linear mapping $$\:{W}_{h}$$, and passed through an activation function $\phi$ (e.g. ReLU) to produce a unified hidden representation. Finally, a linear output layer (weight $W_0$ and bias $b_0$) maps this representation to the predicted UTS. The overall mapping of the Adaptive Multiscale Network can be summarized in a single formulation in Eq. (7)^34^.

7 $$\:\widehat{y}={W}_{0}.\varphi\:\left({W}_{h}.ReLU\left(BN\left(\left[\mathrm{tanh}\left(B{N}_{1}\left({W}_{1}x\right)\right);ReLU\left(B{N}_{2}\left({W}_{2}X\right)\right);LeakyReLU\left(B{N}_{3}\left({W}_{3}x\right)\right)\right]\right)\right)\right)+{b}_{0}$$

This equation compactly represents the multiscale feature extraction (inside the brackets) and the subsequent fusion into a final prediction. In simpler terms, the network is adapting to multiple feature scales: for example, the $\tanh$ branch can attenuate extreme input values (simulating an effect of diminishing returns at high pressures or times), whereas the leaky ReLU branch allows linear extrapolation even for large inputs (capturing scenarios where increasing a parameter continues to strengthen the weld). By learning the appropriate combination of these branches, the model can handle a variety of relationships within the data from nearly linear to strongly nonlinear – which is particularly useful given the mixed nature of effects in friction welding (e.g., some factors may have linear influence on UTS up to a point, beyond which the effect saturates or even reverses). The Adaptive Multiscale Network’s fused representation is then passed through a final linear layer to output the UTS prediction. This architecture provides a robust approach to model the welding process as it captures diverse patterns in the input features across different magnitudes and regimes, complementing the other two models by focusing on the breadth of feature transformations.

##### Model training and validation

All three deep learning models were trained to predict UTS from the input welding parameters using a supervised regression strategy. The mean squared error (MSE) loss was minimized via the Adam optimizer (learning rate = 0.001) to approximate the nonlinear relationship between process parameters and UTS. Given the small dataset (18 samples), a 70:30 train validation split was applied in each run, ensuring representation of both faying geometries and parameter ranges. Hyperparameters such as epochs (max. 200–300), learning rate, and hidden layer size were conservatively tuned to prevent overfitting, with early stopping triggered by rising validation loss. During training, loss decreased rapidly in the initial epochs and then stabilized, with validation loss remaining close to training loss, indicating good generalization. Model performance was evaluated on the validation set using R^2^, MSE, and mean absolute error (MAE), along with scatter plots of predicted versus actual UTS.

## Results and discussion

This section presents the experimental results and machine learning-based predictions for the AA2024-T351 aluminum alloy welded using Direct Drive Friction Welding (DDFW) under various process parameter settings. Table 4 summarizes the measured tensile and torsional strengths of the weld joints obtained under different welding conditions.

**Table 4:** Experimental layout and its results.

Exp. no.	Faying geometry (type)	Friction pressure (MPa)	Forging pressure (MPa)	Rotational speed (rpm)	Friction time (min)	UTS (MPa)	Torsion (MPa)
1	Drilled-to-bossed	20	30	1800	2	281	298.91
2	Drilled-to-bossed	20	50	2000	3	478	312.42
3	Drilled-to-bossed	20	70	2200	4	508	318.24
4	Drilled-to-bossed	30	30	1800	3	335	293.55
5	Drilled-to-bossed	30	50	2000	4	522	320.34
6	Drilled-to-bossed	30	70	2200	2	540	328.38
7	Drilled-to-bossed	40	30	2000	2	349	159.15
8	Drilled-to-bossed	40	50	2200	3	512	325.56
9	Drilled-to-bossed	40	70	1800	4	505	328.70
10	Flat-to-flat	20	30	2200	4	394	218.10
11	Flat-to-flat	20	50	1800	2	426	206.31
12	Flat-to-flat	20	70	2000	3	450	278.23
13	Flat-to-flat	30	30	2000	4	386	298.52
14	Flat-to-flat	30	50	2200	2	501	310.50
15	Flat-to-flat	30	70	1800	3	415	308.98
16	Flat-to-flat	40	30	2200	3	338	268.75
17	Flat-to-flat	40	50	1800	4	402	208.67
18	Flat-to-flat	40	70	2000	2	465	242.86

### Influence of process parameters on tensile strength

In this study, a total of 18 experiments were conducted using direct drive friction welding (DDFW) to explore the influence of key process parameters on the tensile strength of AA2024-T351 aluminum alloy joints. The parameters under investigation included faying surface geometry, friction pressure, forging pressure, rotational speed, and friction time, systematically varied according to an L_18_ orthogonal array. The experimental results revealed a wide variation in ultimate tensile strength (UTS), ranging from 281 MPa to 540 MPa, highlighting the significant role of parameter selection in determining weld quality. The maximum tensile strength of 540 MPa was observed in Experiment 6, which employed a drilled-to-bossed faying geometry, 30 MPa friction pressure, 70 MPa forging pressure, 2200 rpm rotational speed, and 2 min of friction time. This optimal combination suggests that a moderately high friction pressure, coupled with a high forging force and elevated rotational speed, promotes sufficient interfacial heat generation and material plasticization, resulting in enhanced metallurgical bonding. Conversely, the lowest tensile strength of 281 MPa occurred in Experiment 1, which also used a drilled-to-bossed geometry but with lower friction and forging pressures (20 MPa and 30 MPa, respectively), 1800 rpm rotational speed, and the same 2-minute friction time. The lower UTS in this case can be attributed to inadequate heat input and insufficient plastic deformation at the weld interface, leading to suboptimal joint formation. A comparison between the two faying surface geometries, drilled-to-bossed and flat-to-flat, reveals that the drilled-to-bossed configuration consistently delivered superior tensile strength across various parameter combinations. This improvement is likely due to the mechanical interlocking effect and increased surface area at the weld interface, which enhances bonding and load transfer capability. Among the process parameters, forging pressure and rotational speed exhibited the most pronounced influence on tensile strength. Higher forging pressures (particularly 70 MPa) facilitated improved consolidation of the softened material, while increased rotational speeds (especially 2200 rpm) enhanced frictional heating and softened the material at the interface for better fusion. Friction pressure also contributed positively, although to a lesser extent, with moderate levels (30–40 MPa) proving more effective than the lowest setting. Interestingly, friction time showed a non-linear effect: while prolonged time can promote bonding, excessive durations may lead to overheating and degradation of mechanical properties. The best results were achieved at 2 to 3 min, depending on the pressure and speed conditions. In summary, the experimental findings underscore the importance of synergistically tuning process parameters in DDFW. The use of a drilled-to-bossed geometry, in combination with high forging pressure, elevated rotational speed, and optimized friction time, was found to significantly enhance the tensile strength of friction-welded AA2024-T351 aluminum alloy joints.

### Influence of process parameters on torsional strength

In addition to tensile performance, torsional strength was evaluated to assess the joints’ resistance to twisting, an essential property for components subjected to rotational or shear loading. The experimental results, summarized in Table 4, revealed torsional strength values ranging from 159.15 MPa to 328.70 MPa, highlighting the significant impact of process parameters. The highest torsional strength (328.70 MPa) was obtained in experiment 9, which employed a drilled-to-bossed geometry, 40 MPa friction pressure, 70 MPa forging pressure, 1800 rpm rotational speed, and 4 min of friction time. This combination facilitated effective plastic deformation and interfacial bonding, minimizing void formation and ensuring structural integrity. In contrast, the lowest torsional strength (159.15 MPa) was recorded in experiment 7, where lower forging pressure (30 MPa) and shorter friction time (2 min) limited material consolidation, despite the same faying geometry and a relatively high friction pressure. The drilled-to-bossed configuration consistently outperformed the flat-to-flat geometry across all experiments. This improvement is primarily attributed to its enhanced mechanical interlocking mechanism, which significantly increases resistance to shear and rotational displacement. The male-female interface extends the effective contact area and distributes stresses more uniformly along the weld, reducing the likelihood of slippage or premature failure. Furthermore, the protruding boss facilitates better axial alignment during the friction phase, resulting in more uniform heat generation and plastic flow, while the cavity in the female specimen helps localize heat and pressure, promoting plastic deformation and grain refinement. These geometric and thermal advantages synergistically improve weld strength and reliability, particularly under torsional loads. Building upon these observations, a detailed analysis of individual parameters reveals that forging pressure emerged as the most influential factor. A forging pressure of 70 MPa consistently yielded stronger joints due to enhanced material consolidation under high compressive force. Friction pressure and rotational speed also contributed significantly friction pressures around 40 MPa ensured adequate heat input for plasticization, while rotational speeds between 1800 and 2200 rpm facilitated uniform thermal distribution and minimized interfacial defects. Friction time exhibited a nonlinear effect: while longer durations, such as 4 min, improved strength under optimal pressure conditions, excessive friction time did not consistently enhance performance, especially when combined with suboptimal forging force. Overall, the findings demonstrate that high forging pressure, longer friction time, and the adoption of a drilled-to-bossed interface are key enablers for maximizing torsional strength in DDFW of AA2024-T351 aluminum alloy, offering critical insights for improving the reliability of friction-welded joints in shear-critical applications.

### Deep learning algorithms prediction

**Fig. 6:**
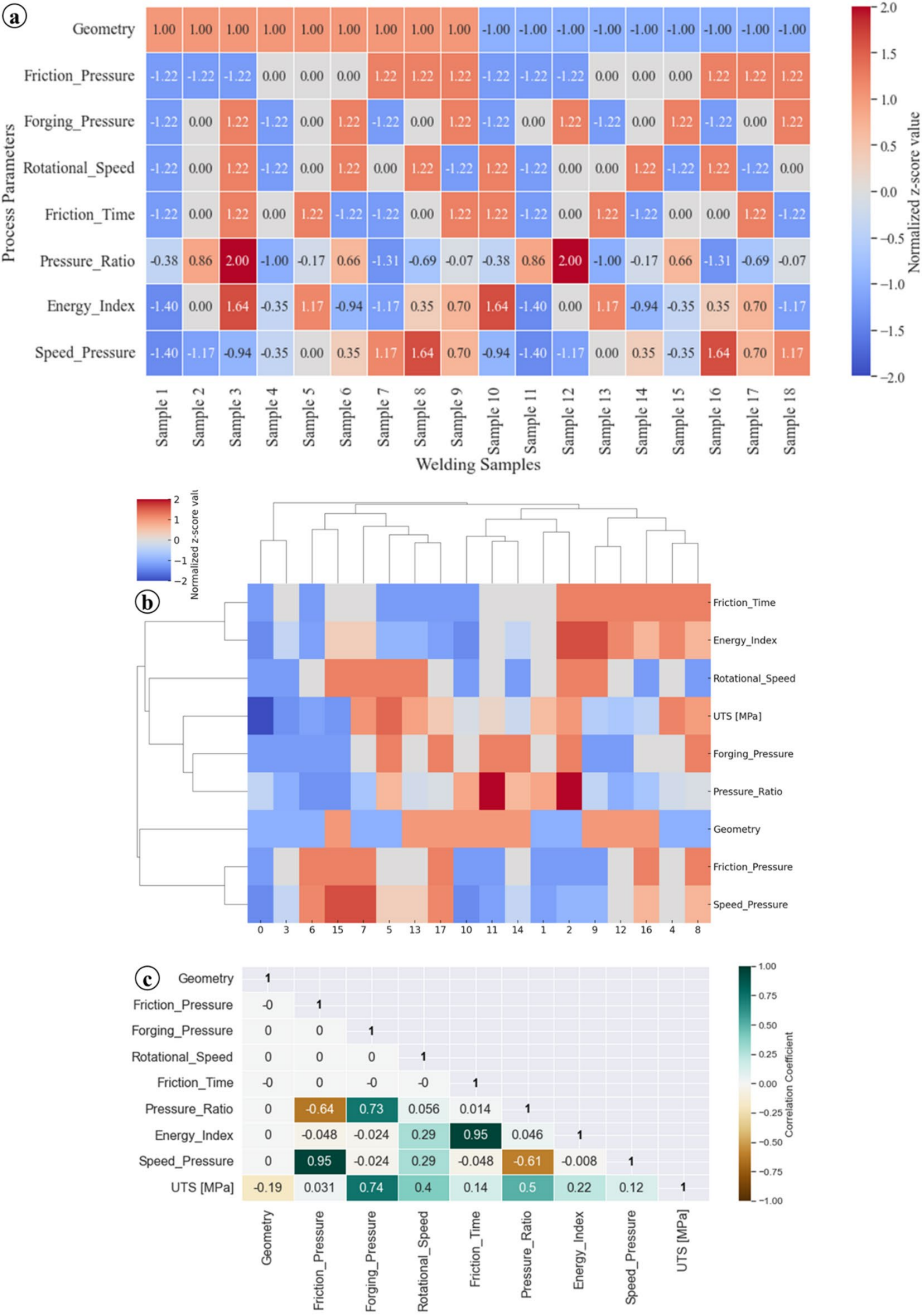
Visualization and correlation analysis plot of welding process parameters and UTS: **a** normalized z-score heatmap of process parameters across welding samples, **b** hierarchical clustering heatmap revealing similarity patterns among parameters and samples, and **c** correlation matrix between process parameters and ultimate tensile strength (UTS).

**Fig. 7:**
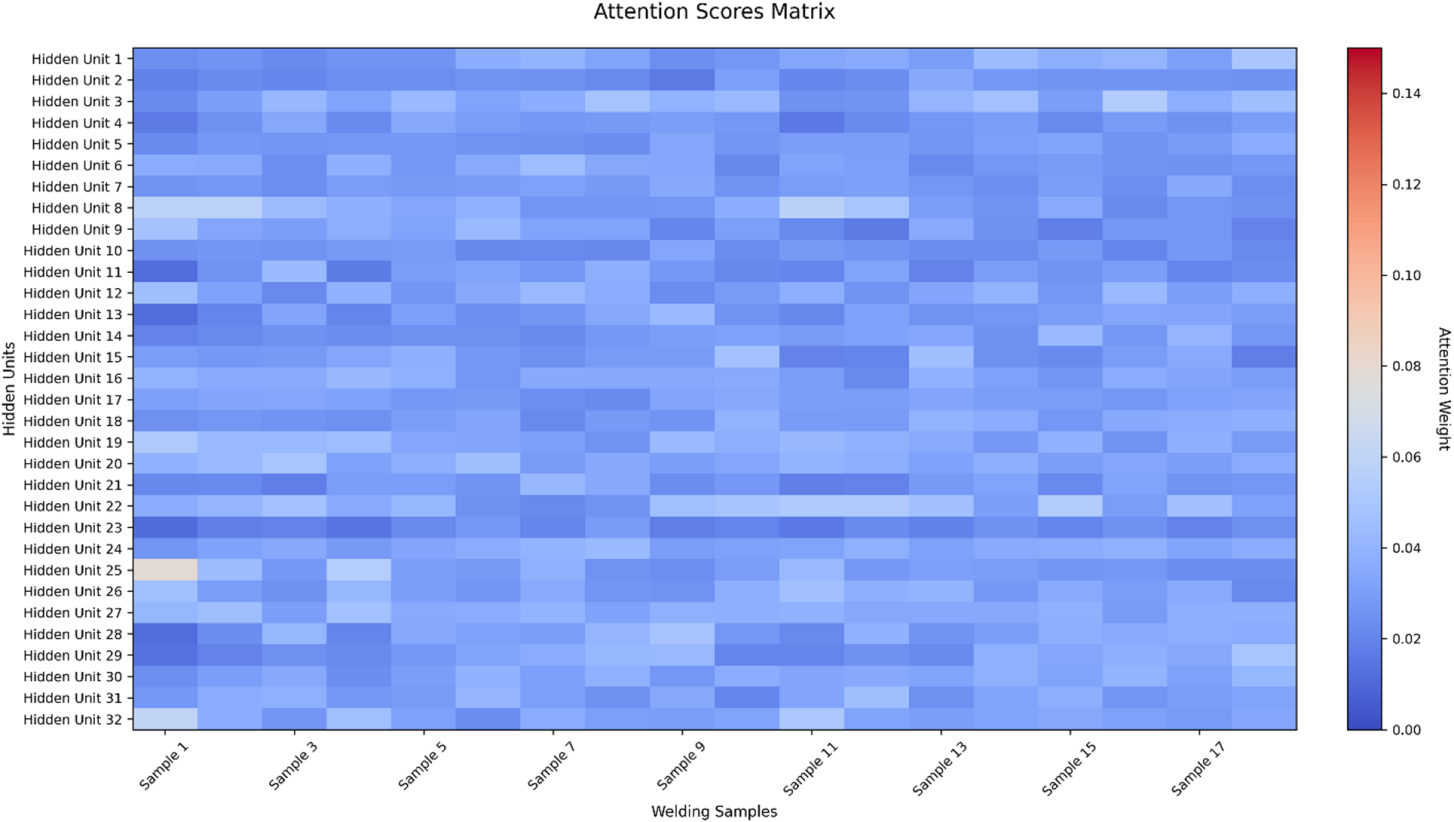
Attention scores computed by the compact attention mechanism. The heatmap displays the softmax-normalized attention weights assigned to each hidden unit for every sample, indicating which feature representations the model focuses on during prediction.

**Fig. 8:**
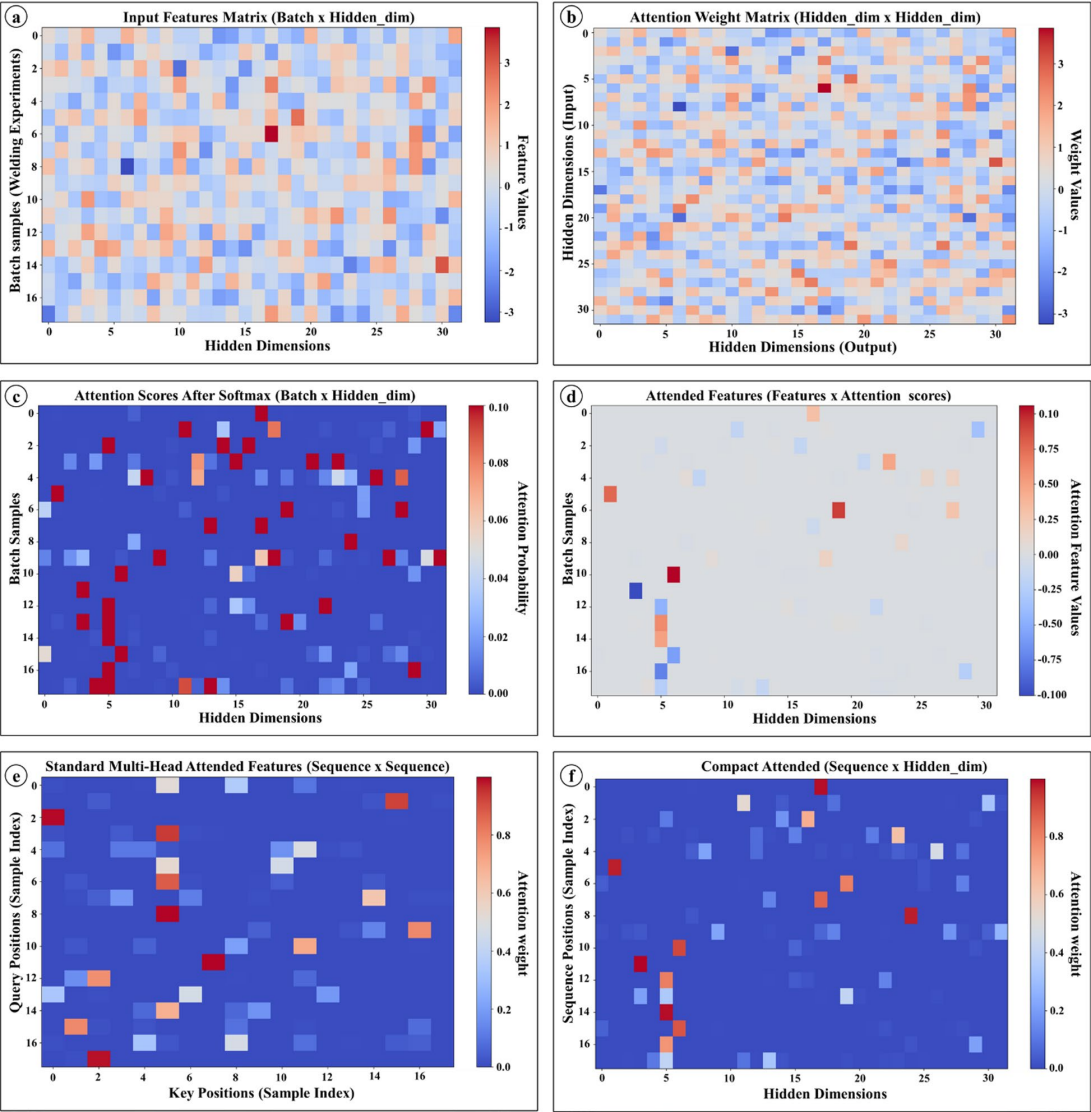
Mathematical operations and architectural comparison in compact attention mechanism. **a** Input features matrix (18 samples × 32 hidden dimensions), **b** learned attention weight matrix (32 × 32), **c** attention scores after softmax normalization revealing sparse, **d** element-wise multiplication **e** standard multi-head attention, **f** compact attention.

The normalized z-score plot in Fig. 6a compares multiple welding process parameters across 18 experimental runs, enabling direct comparison of variables with different units and scales. Samples 3 and 12 exhibit pronounced peaks in several parameters, potentially indicating optimal conditions or extreme stress states. Notably, the speed pressure parameter mirrors the energy index pattern in several instances, reinforcing that it is jointly influenced by rotational speed and friction pressure. The cyclic patterns in Friction Pressure, Forging Pressure, and Friction Time suggest a designed experimental matrix rather than random variation. To further understand operational regimes trends, Fig. 6b shows a clustered heatmap, where row clustering groups process parameters with similar z-score patterns. For instance, energy Index and Friction Time are closely grouped, suggesting that longer friction times are linked with higher energy input. Figure 6c represents the pairwise Pearson correlation coefficients among welding process parameters and the ultimate tensile strength (UTS), which serves as the primary weld quality metric. The geometry feature shows a clear binary pattern, with the first 9 samples (red/orange region) representing drilled-to-bossed geometry and the remaining 9 samples (blue region) corresponding to flat-to-flat geometry. This distinct separation demonstrates how the categorical encoding effectively captures the two different welding configurations in the dataset. The process parameters exhibit significant variability across samples, which is essential for training a robust attention mechanism. Friction pressure shows alternating patterns of low (blue), medium (neutral), and high (red) values, reflecting the systematic experimental design with pressures ranging from 20 to 40 MPa. Similarly, forging pressure displays a more complex pattern with broader ranges (30–70 MPa), indicating the substantial influence this parameter has on the welding process. The rotational speed parameter shows moderate variation around the normalized center, while friction time demonstrates clear discrete levels corresponding to the 2–4 min experimental ranges. The engineered interaction features reveal important process relationships. This suggests these samples represent conditions where forging pressure significantly exceeds friction pressure, which could be critical for UTS prediction. The energy index feature shows strong correlations with both friction pressure and time, as expected from its mathematical definition, while the speed-pressure interaction feature captures the combined effects of rotational speed and friction pressure. From an attention mechanism perspective, this feature distribution is highly favorable for learning meaningful patterns. The clear geometric separation allows the attention weights to distinguish between different welding configurations, while the systematic variation in process parameters provides sufficient diversity for the model to learn parameter-UTS relationships. The presence of both positive and negative normalized values across all features ensures that the attention mechanism can effectively weight the importance of different parameter combinations. The engineered features add valuable non-linear interactions that help the compact attention network capture complex relationships that might not be apparent from individual parameters alone, ultimately enabling more accurate UTS predictions through focused attention on the most relevant feature combinations for each welding sample. Next, instead of traditional (multi-head) attention that considers all possible pairwise interactions (which is computationally expensive for small datasets), this compact attention approach learns an attention score for each hidden feature dimension by passing the features through another linear layer, followed by a softmax normalization. These attention scores indicate the relative importance of each hidden feature for the final prediction. A compact attention mechanism calculates attention weights for the feature representation as shown in Eq. (2). The attention scores matrix shown in Fig. 7 demonstrates the learned feature prioritization within the compact attention architecture when applied to friction welding parameters. The distribution pattern exhibits a relatively uniform attention allocation across the 32 hidden units, with weights predominantly spanning 0.02–0.06, which suggests effective regularization through the softmax normalization. This finding contradicts typical attention mechanisms that often exhibit sparse activation patterns, indicating that the compact formulation promotes distributed feature representation rather than selective attention peaks. Examination of unit-specific activation patterns reveals several noteworthy observations. Hidden Units 21–23 display elevated attention coefficients for specific sample subsets, particularly samples 1–3 and 15–16 for Unit 21, which correspond to drilled-to-bossed geometries under low friction pressure conditions (20 MPa). This selective activation suggests that certain hidden units have specialized to detect specific parameter combinations that may be critical for UTS determination. The consistent activation pattern across geometrically similar samples indicates that the attention mechanism has learned to encode geometry-dependent features despite the relatively small dataset size. The transition region between samples 9–10 marks a clear shift in attention dynamics, corresponding to the change from drilled-to-bossed to flat-to-flat geometry configurations. Multiple hidden units (8, 14, 25) exhibit discontinuous attention patterns across this boundary, suggesting that the model has successfully learned to differentiate between the two welding geometries at the feature level. This geometric sensitivity is particularly significant given that the geometry encoding represents only one of eight input features, yet its influence propagates through the hidden representation space in a measurable way. The absence of extreme attention values (> 0.15) across all samples indicates stable training convergence and suggests that the model is not overfitting to particular samples or parameter combinations. This stability is crucial for generalization, especially considering the limited training data (*n* = 18). The distributed attention pattern also implies that UTS prediction relies on complex parameter interactions rather than individual parameter dominance, which aligns with the known metallurgical complexity of friction welding processes, where multiple thermal, mechanical, and geometric factors simultaneously influence joint strength. The model then performs an elementwise multiplication between the attention scores and the feature representation, effectively reweighting features according to their learned relevance shown in Eq. (3). This “attended” feature vector is then fed through a regression head composed of several fully connected layers, ultimately producing a single output i.e. the predicted UTS value shown in Eq. (7). Figure 8a illustrates the input feature matrix after linear transformation into the hidden dimensional space, where the 18 welding experiments are mapped to a 32-dimensional hidden representation. The feature distribution exhibits a balanced mix of positive and negative activations across the hidden dimensions, with notable clustering patterns that suggest the linear transformation has successfully captured meaningful parameter relationships. The absence of extreme outliers (values beyond ± 3) indicates stable feature extraction without gradient explosion issues, while the heterogeneous activation patterns across samples demonstrate that the transformation preserves sample-specific characteristics rather than converging to uniform representations. The attention weight matrix (Fig. 8b) reveals the learned parameter relationships within the hidden space, displaying a complex interaction network where each hidden dimension influences every other dimension through trainable weights. The matrix exhibits a relatively balanced structure with moderate positive and negative connections, suggesting that the attention mechanism has learned sophisticated feature interdependencies rather than simple linear combinations. The presence of both strong positive (red) and negative (blue) connections indicates that the mechanism can both amplify and suppress specific feature combinations, enabling nuanced attention allocation based on parameter contexts. Figure 8c demonstrates the critical softmax normalization step, where raw attention logits are converted to probability distributions that sum to unity across each sample’s hidden dimensions. The sparse activation pattern, with most probabilities concentrated below 0.04, reveals that the attention mechanism has learned to focus selectively on specific hidden units rather than distributing attention uniformly. Notable attention peaks (red regions) appear at specific sample-dimension combinations, particularly around samples 0, 5, 10, and 15, suggesting that certain welding parameter configurations trigger focused attention responses that may correlate with critical UTS-determining conditions. The final attended features (Fig. 8d) show the element-wise multiplication of original features with attention scores, producing the weighted representation that feeds into the output layers. The resulting feature matrix exhibits enhanced contrast compared to the input features, with attention-amplified regions showing stronger activations while suppressed regions approach zero. This selective amplification is particularly evident in the concentrated activation patterns around samples 5, 10, and 15, where high attention scores have effectively highlighted the most relevant feature combinations for UTS prediction.

**Fig. 9:**
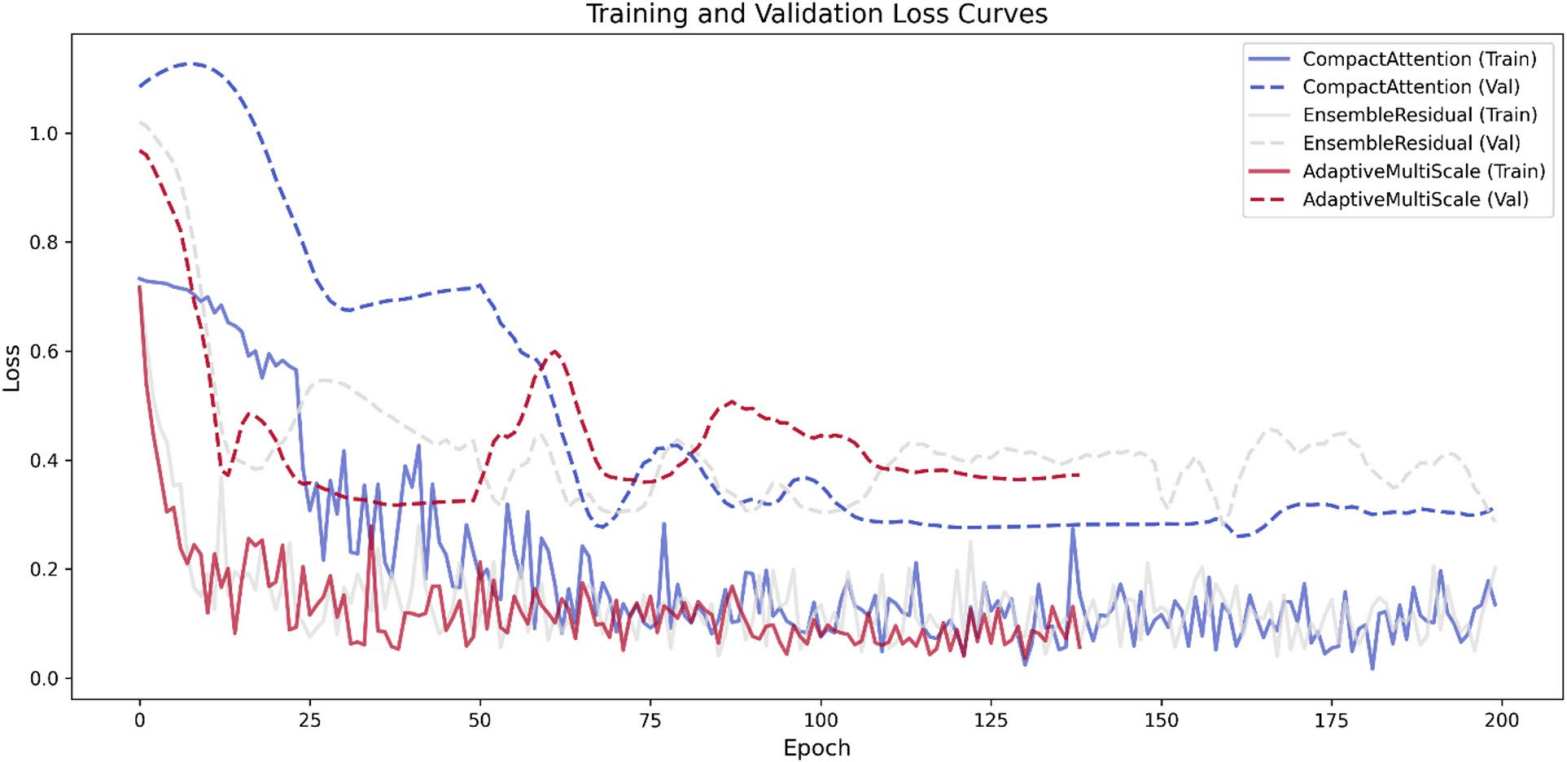
Training and validation loss curves of 200 epochs with early stopping for three deep learning architectures predicting ultimate tensile strength (UTS).

The loss curves reveal distinct training dynamics across the three models. CompactAttention demonstrates rapid initial convergence with both training and validation losses dropping sharply in the first 25 epochs, then stabilizing around 0.3 for training and slightly higher for validation. This pattern suggests efficient learning without significant overfitting. EnsembleResidual shows more volatile training behavior with fluctuating losses throughout the training process, though it eventually achieves good convergence. The validation loss remains relatively stable around 0.4–0.5, indicating reasonable generalization. AdaptiveMultiScale exhibits the most complex training dynamics with multiple phases of convergence and divergence, particularly notable spikes around epochs 60–80, before finally stabilizing. The higher final validation loss compared to training loss suggests some degree of overfitting (Fig. 9).

**Fig. 10:**
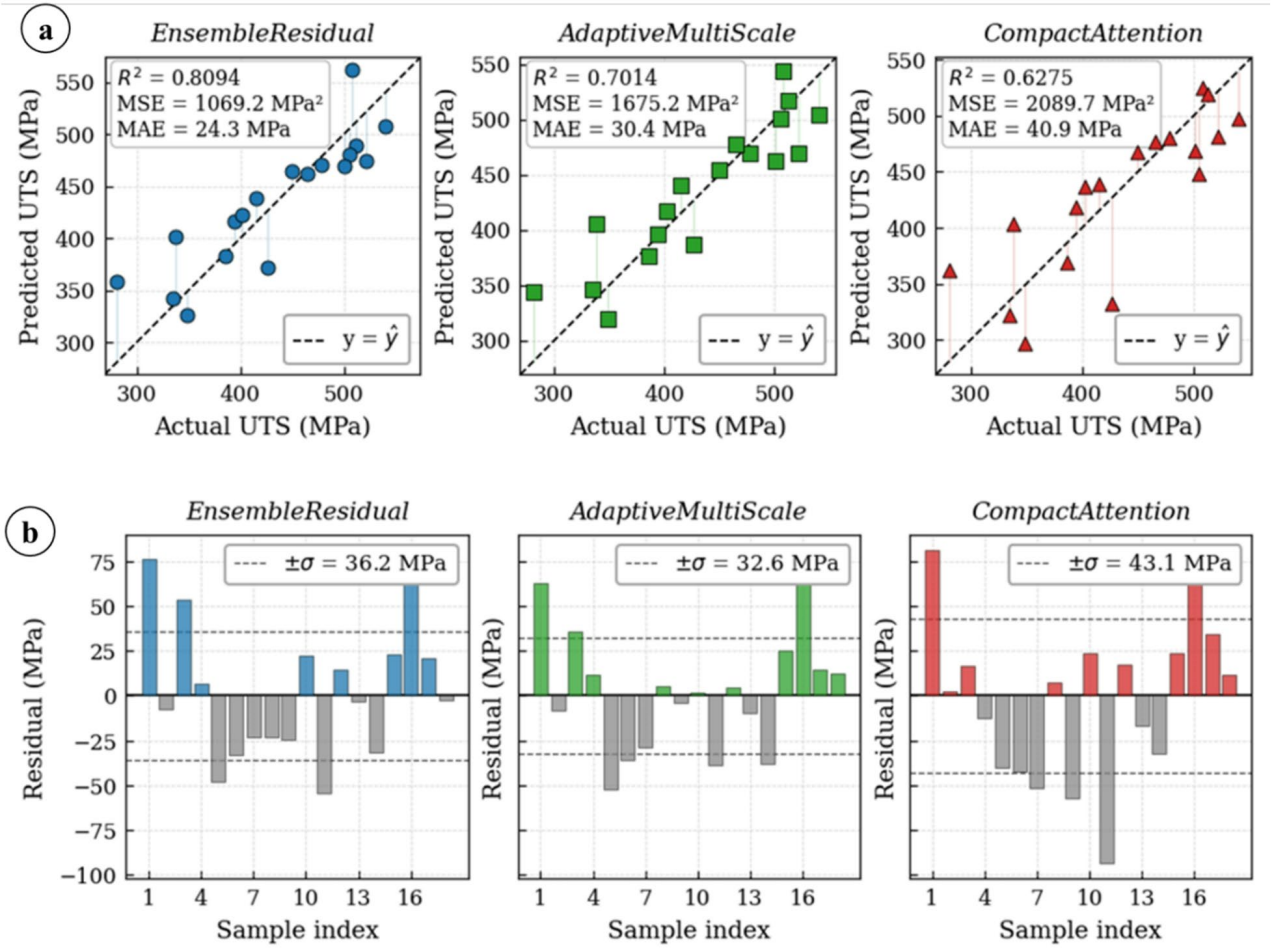
LOOCV-based prediction performance of the three deep learning models for UTS estimation (*n* = 18): **a** scatter plots of actual versus predicted UTS values for EnsembleResidual, AdaptiveMultiScale, and CompactAttention models, with the dashed line representing the ideal prediction (y = $\hat{\mathrm{y}}$) and inset metrics reporting R², MSE, and MAE; **b** per-sample residual distributions across all 18 LOOCV folds, with dashed horizontal lines denoting the ± one standard deviation (±σ) bounds for each model.

Figure 10a presents the actual versus predicted UTS values for the three deep learning models evaluated under LOOCV. The EnsembleResidual model demonstrates the strongest predictive agreement with an R² of 0.8094, MSE of 1069.2 MPa², and MAE of 24.3 MPa, with data points clustering closely around the ideal y = ŷ line. The AdaptiveMultiScale model achieves moderate agreement (R² = 0.7014, MAE = 30.4 MPa), while the CompactAttention model shows the greatest scatter from the diagonal (R² = 0.6275, MAE = 40.9 MPa), indicating comparatively weaker generalization for this small dataset. Figure 10b illustrates the per-sample residuals (predicted − actual) across all 18 LOOCV folds. The EnsembleResidual model exhibits residuals predominantly within ± σ = 36.2 MPa, with only a few outlying samples, reflecting reasonably balanced over- and under-prediction. The AdaptiveMultiScale model shows a tighter standard deviation of ± 32.6 MPa, though several samples display systematic underprediction. The CompactAttention model yields the largest spread (±σ = 43.1 MPa) with notable negative residuals at multiple sample indices, suggesting this architecture struggles to capture the non-linear process–property relationships inherent to the rotary friction welding dataset at *n* = 18.

**Fig. 11:**
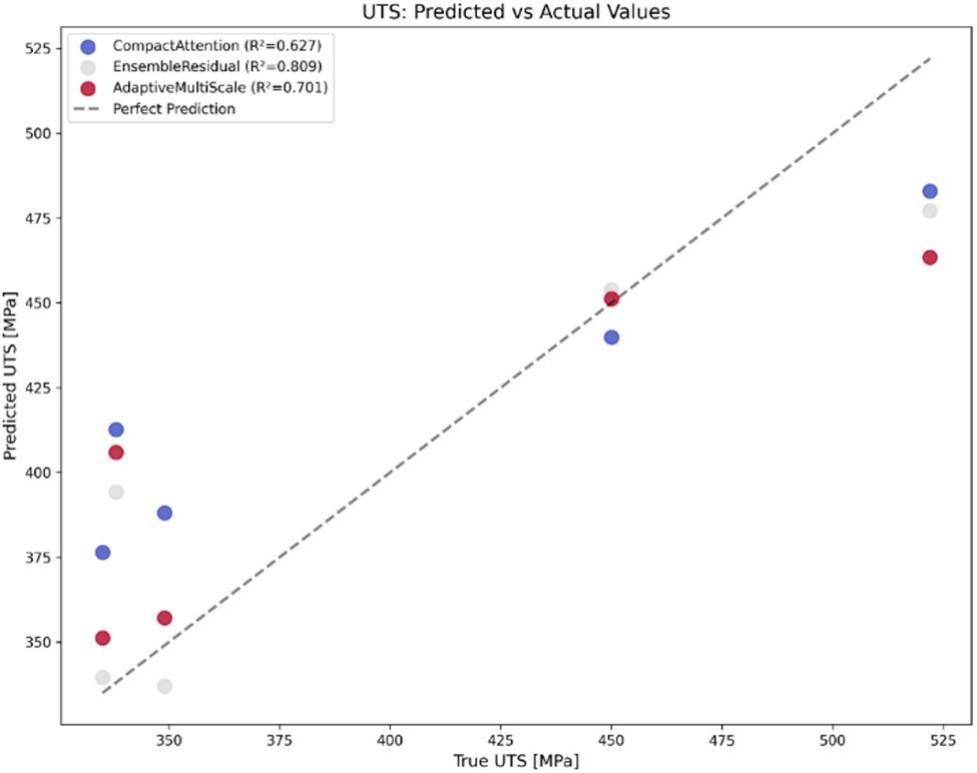
Scatter plot comparison of predicted versus actual ultimate tensile strength (UTS) values for three neural network models, with the diagonal dashed line representing fitted line.

The scatter plot analysis shown in Fig. 11 reveals EnsembleResidual as the clear winner with an R² of 0.809, showing data points closely aligned with the perfect prediction line across the entire UTS range from 330 to 520 MPa. The model demonstrates consistent accuracy for both low and high UTS values. AdaptiveMultiScale achieves a respectable R² of 0.701, with most predictions falling reasonably close to the ideal line, though with slightly more scatter than EnsembleResidual. CompactAttention shows the weakest correlation at R² = 0.627, with noticeable deviations from the perfect prediction line, particularly for extreme values. The spread of data points indicates less reliable predictions across the UTS spectrum.

**Fig. 12:**
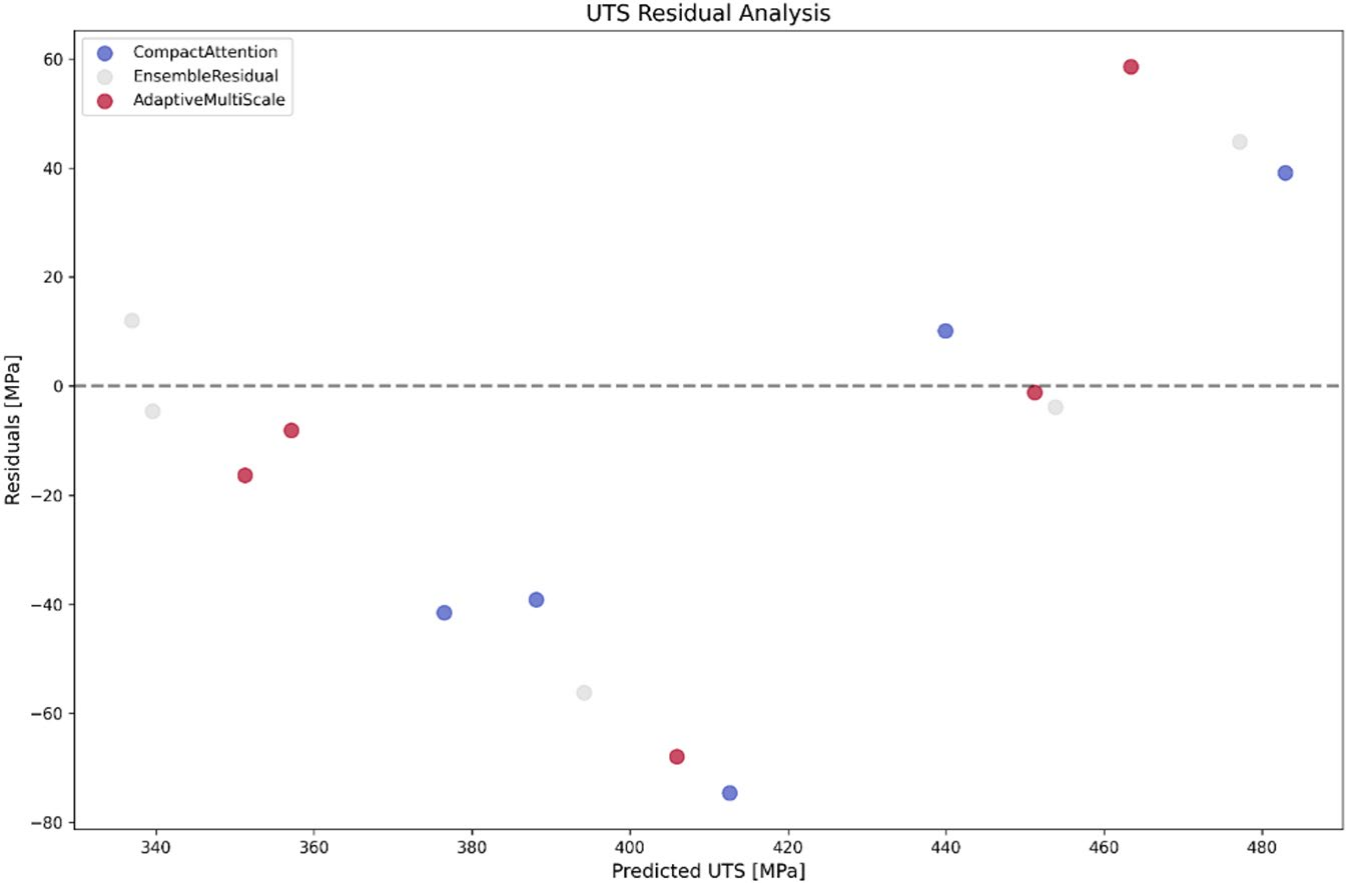
Residual analysis plot showing the distribution of prediction errors (actual minus predicted UTS values) as a function of predicted UTS for model bias assessment. The horizontal dashed line at zero represents perfect prediction accuracy.

**Fig. 13:**
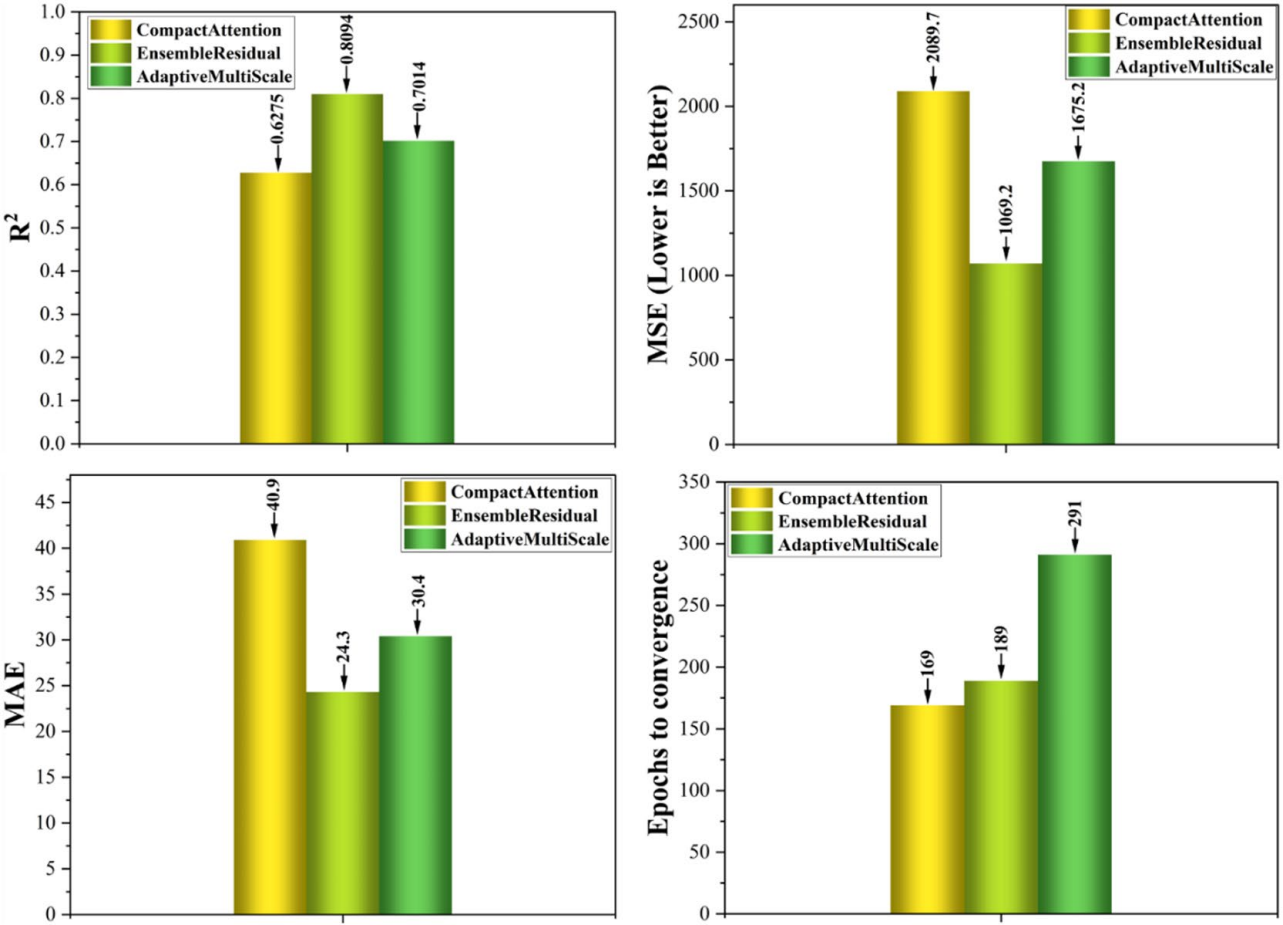
Performance comparison of three neural network architectures across four key evaluation metrics.

The residual plots shown in Fig. 12 provide insight into prediction bias patterns. EnsembleResidual exhibits the most balanced residual distribution with points scattered relatively evenly above and below the zero line, though with some concentration of negative residuals around 400 MPa predicted UTS. CompactAttention shows a systematic bias with predominantly negative residuals for lower predicted values and positive residuals for higher values, suggesting the model tends to underpredict low UTS and overpredict high UTS values. AdaptiveMultiScale displays mixed residual patterns with both positive and negative outliers, including some significant deviations exceeding ± 60 MPa, indicating inconsistent prediction reliability across different UTS ranges. The comparative analysis shown in Fig. 13 confirms EnsembleResidual’s superior performance across multiple metrics. With the highest R² score (0.8094) and lowest MSE (1069.2) and MAE (24.3), it demonstrates the best overall predictive accuracy and precision. The training efficiency of 189 epochs to convergence represents a reasonable balance between thoroughness and computational efficiency. CompactAttention, while showing the fastest convergence at 169 epochs, sacrifices accuracy with the lowest R² (0.6275) and highest error metrics (MSE: 2089.7, MAE: 40.9). AdaptiveMultiScale falls between the two with moderate accuracy metrics (R² = 0.7014, MSE: 1675.2, MAE: 30.4) but requires the longest training time at 291 epochs, suggesting computational inefficiency despite reasonable final performance.

## Conclusion

This study confirms that proper selection of DDFW parameters, coupled with an improved joint design, can markedly enhance the mechanical properties of AA2024-T351 welds. The novel drilled-to-bossed configuration was found to consistently outperform the conventional flat-to-flat interface, yielding higher tensile and torsional strengths due to superior mechanical interlocking and an expanded bonding area. Among the process parameters examined, forging pressure and rotational speed emerged as the most influential factors governing joint strength. Specifically, a moderate friction pressure (30 MPa) paired with a high forging pressure (70 MPa) and elevated spindle speed (2200 rpm) produced the maximum observed tensile strength (540 MPa), effectively matching the base material’s tensile capacity. In contrast, attaining the highest torsional strength (329 MPa) required a slightly higher friction pressure (40 MPa) and prolonged friction time (4 min) at the same forging pressure (70 MPa), indicating that extended frictional heating and plastic deformation under sufficient pressure can improve resistance to shear loads. These findings underscore the need for adequate forging force to consolidate the weld and sufficient spindle speed to generate the thermal conditions for robust bonding, while also highlighting that friction time must be carefully optimized to avoid thermal degradation of joint properties. Additionally, the application of deep learning models demonstrated considerable promise in predicting weld outcomes from process parameters. Among the architectures evaluated, the EnsembleResidualNet model achieved the highest predictive accuracy (R² ≈ 0.81), outperforming the CompactAttention (R² ≈ 0.63) and AdaptiveMultiScale (R² ≈ 0.70) networks in forecasting ultimate tensile strength. The superior performance of this ensemble residual network highlights the benefit of combining multiple learning pathways to capture the complex, non-linear relationships inherent in friction welding processes. By integrating data-driven predictive tools with experimental optimization, manufacturers can better anticipate joint performance and refine process parameters virtually, reducing the need for extensive trial-and-error. Overall, the convergence of optimized DDFW parameters, an enhanced faying surface design, and machine-learning-driven predictions provides a robust strategy for producing high-strength, reliable AA2024-T351 joints. These insights carry significant implications for aerospace and automotive engineering, where improved joint efficiency and strength directly contribute to safer, lighter structural components and enhanced fuel efficiency in vehicles and aircraft.

Future work will include microstructural characterization (optical microscopy, SEM, and grain size analysis) and in-situ temperature monitoring to experimentally verify the proposed mechanisms.

## Data Availability

The datasets used and analysed during the current study available from the corresponding author on reasonable request.
